# Superficial temporal artery–middle cerebral artery bypass in combination with encephalo-myo-synangiosis in Chinese adult patients with moyamoya disease

**DOI:** 10.3389/fsurg.2023.1100901

**Published:** 2023-01-24

**Authors:** Lu Li, Anji Wang, Changhui Wang, Hanbin Zhang, Deshen Wu, Guangliang Zhuang, Jie Wang

**Affiliations:** ^1^ Department of Neurosurgery, Shanghai Deji Hospital, Shanghai, China; ^2^ Epilepsy Center, Shanghai Deji Hospital, Shanghai, China

**Keywords:** moyamoya disease, direct revascularisation technique, STA-MCA bypass, indirect revascularisation technique, cerebral blood flow

## Abstract

**Objective:**

To evaluate the feasibility and safety of superficial temporal artery (STA)–middle cerebral artery (MCA) anastomosis in combination with encephalo-myo-synangiosis (EMS) in Chinese adult patients with moyamoya disease (MMD).

**Methods:**

A total of 65 patients with MMD who underwent combined STA–MCA bypass + EMS surgical revascularisation were included in this study. Each patient had a follow-up visit 6 months after discharge. Early bypass function was evaluated *via* computed tomography angiography and digital subtraction angiography, which were performed preoperatively and at 6 months after surgery. The perfusion parameters of cerebral blood flow (CBF), cerebral blood volume (CBV), mean transit time (MTT) and time to peak (TTP) were obtained and analysed. The clinical status of each patient was evaluated using a modified Rankin scale (mRS) preoperatively and at 1 week and 6 months after surgery.

**Results:**

Among the 65 enrolled patients, postoperative complications were observed in 5 (7.69%) patients, with 2 cases of dysphasia, 2 cases of new cerebral infarction and 1 case of seizure. Six months after surgery, 66 out of 68 hemispheres were found to have a functioning extra-intracranial bypass, and the patency rate was 97.06%. In terms of CBF perfusion, both the CBF and CBV increased significantly, while the MTT and TTP decreased after surgery. The mRS scores measured 1 week and 6 months after surgery were much lower than those measured preoperatively.

**Conclusion:**

A direct STA–MCA bypass procedure in combination with indirect EMS bypass is feasible and safe for Chinese adult patients with MMD.

## Introduction

Moyamoya (Japanese for “puff of smoke”) disease (MMD), which was first described by Japanese neurosurgeons, is a chronic occlusive cerebrovascular disorder. It is characterised by progressive stenosis or occlusion of the intracranial portion of the internal carotid artery and its proximal branches (1, 2). The incidence of MMD is higher in East Asian countries, such as Japan, China and Korea, than in other countries (3). It has been reported that the incidence of MMD in Japan is 5.22–10.50 per 100,000 people (4). However, data on MMD in China are scarce. The clinical symptoms of MMD are caused mainly by cerebral ischaemia or haemorrhage. If patients with MMD are left untreated, the natural prognosis is generally unfavourable (5).

Currently, surgical revascularisation plays an important role in the management of MMD (5). The main goal of surgery is to improve blood flow to the hypoperfused brain (6). Different revascularisation strategies to attenuate ischaemic insults have been reported previously; these include direct bypasses, such as superficial temporal artery (STA)–middle cerebral artery (MCA) anastomosis (7) and STA–anterior cerebral artery anastomosis (8); indirect bypasses, such as encephalo-duro-arterio-synangiosis (EDAS) (9) and encephalo-myo-synangiosis (EMS) (10), and combinations of direct and indirect anastomosis (11). However, the optimal revascularisation strategy is still debated.

Combined direct and indirect cerebral revascularisation has some advantages over surgery alone, such as better short-term and long-term outcomes and fewer complications (5, 12). Zhang et al. (12) analysed data from 123 patients with haemorrhagic MMD and demonstrated that combined STA–MCA bypass + EDAS was superior to EDAS alone in terms of preventing rebleeding events. Czabanka et al. (5) enrolled 37 patients with MMD and found that STA–MCA + EMS was superior to EMS alone in restoring cerebrovascular reserve capacity in adult patients with MMD. Bot et al. (13) also revealed that STA–MCA + EMS was feasible and safe for patients with MMD aged ≤3 years. However, it has not been determined whether the direct STA–MCA bypass procedure in combination with indirect EMS bypass is feasible for Chinese adult patients with MMD.

The advantage of EMS is that the blood supply is derived mainly from the middle and posterior deep frontal arteries, and it has a prolonged effect on increasing blood supply (14). Yongyi et al. (15) demonstrated a low complication rate in adult patients with MMD who were treated with STA–MCA shunts combined with temporalis muscle sticking (i.e., EMS). While EDAS is simple to perform and has few complications, the distal end of the STA is continuous in this procedure, and its application direction is limited by the course of the STA (16). A study of EDAS application in adult patients indicated that 23% of 306 surgeries had good blood supply reconstruction, while it was fair in 53% of cases (17).

In the past, STA–MCA bypass surgery combined with indirect EMS has been used mainly in paediatric patients, who have been the focus of most studies. There are few studies on adult patients, and it is difficult to obtain relevant information on the safety and efficacy of this surgery in adults. Therefore, the novelty of the present study lies in its evaluation of the feasibility and safety of STA–MCA + EMS surgery in Chinese adult patients with MMD. The findings will provide relevant information and be a reference for the surgical treatment of adult patients, leading to better treatment methods and improved quality of life.

## Methods

### Study design and population

This retrospective study was approved by the ethics committee of Shanghai Deji Hospital in accordance with the principles of the Declaration of Helsinki. All participants provided written informed consent to publish their data.

From January to October 2021, 65 patients with MMD who received surgical revascularisation were enrolled in this study. Preoperatively, patients with MMD underwent digital subtraction angiography (DSA). The diagnosis of MMD was based on the diagnostic criteria established by the Research Committee on Spontaneous Occlusion of the Circle of Willis (Moyamoya disease) of the Ministry of Health of Japan (18). The diagnostic criteria for MMD by cerebral angiography were as follows: (1) stenosis/occlusion of the terminal portion of the intracranial internal carotid artery or proximal portions of the anterior artery and/or the MCA, (2) abnormal vascular networks in the vicinity of the occlusive or stenotic lesions in the arterial phase and (3) bilaterality of the above findings (18).

The inclusion criteria were as follows: (1) patients aged ≥18 years and (2) patients who had undergone combined direct and indirect bypass. Patients with systemic diseases causing similar smog-like blood vessels, such as autoimmune disease, meningitis, brain tumours or brain trauma, were excluded. Smog-like disease is characterised by the stenosis and/or occlusion of the end of the internal carotid artery and an abnormal vascular network at the beginning of the anterior cerebral artery and/or the posterior cerebral artery, and it is accompanied by an underlying disease. In unilateral MMD, if an underlying disease is present, it can also be classed as smog-like disease.

### Surgical revascularisation

Surgical indications: (1) a history of transient ischemic attack (TIA), (2) a clear history of cerebral infarction, (3) clear neurological symptoms, such as persistent or intermittent headache, dizziness or disturbance of consciousness and (4) impaired cerebral haemodynamics and no effective compensation, as revealed by angiography.

**Figure 1:**
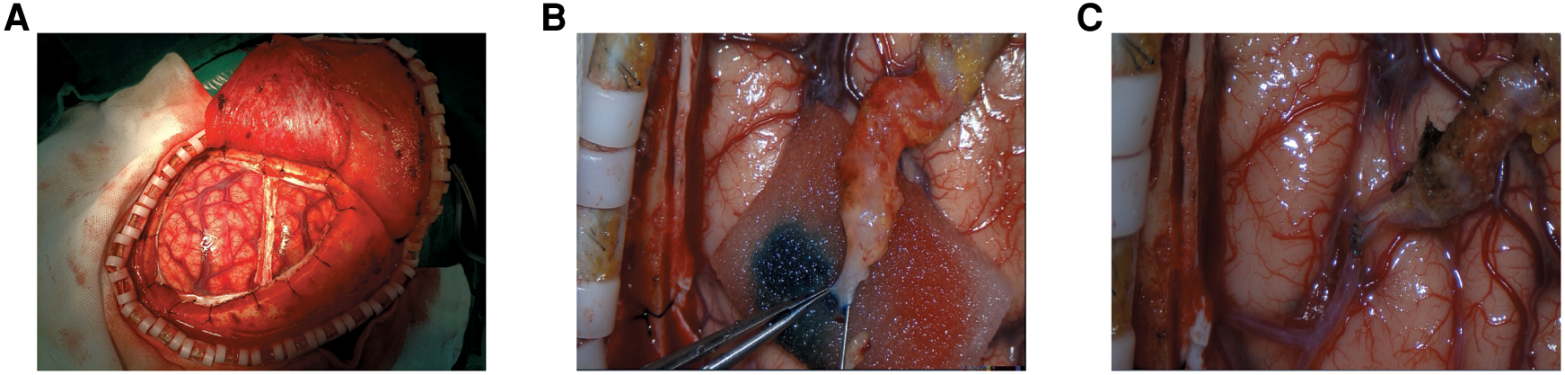
The surgical revascularization procedures. (**A**) The temporal muscle covered by its fascia was incised along the parietal branch; (**B**) The more prominent branch of STA was chosen for the direct anastomosis; (**C**) The STA was fish-mouthed and sutured to a cortical M4 MCA branch with continuous 10-0 sutures.

All the enrolled patients underwent combined (i.e., STA–MCA bypass + EMS) surgical revascularisation. Briefly, the patient's head was fixed by a fixation device under general anaesthesia. The skin was incised, and the temporal muscle covered by its fascia was incised along the parietal branch (Figure 1A). Then, the STA and the artery were dissected meticulously from the surrounding tissue. After the frontal and parietal STA branches were harvested, the most prominent branch was selected for direct anastomosis (Figure 1B). The STA was fish-mouthed and sutured to a rami cortical branch of MCA with continuous 10-0 sutures (Figure 1C). An indocyanine green video angiography assessment was routinely performed intraoperatively to confirm the bypass patency. After completing the direct bypass, indirect EMS was performed. The temporal muscle was placed on the brain surface and sutured to the dural edge. The bone flap was returned and fixed using titanium mini plates. Finally, the skin was closed in a standard fashion.

### Whole-brain computed tomography perfusion

The inspection procedures were as follows (19): A whole-brain computed tomography perfusion (CTP) scan was performed using a 320-slice dynamic volume CT scanner (Aquilion One, Toshiba, Tochigi Pref., Japan) with the following scanning parameters: 80 kV, 150–300 mA, arterial phase tube current = 300 mA, matrix = 512 × 512, slice thickness = 0.5 mm and Z-axis coverage = 16 cm. Here, 50 ml of non-ionic contrast medium (iomeprol: 400 mg I/ml, rate: 6.0 ml/s) and 40 ml of normal saline (rate: 5.5 ml/s) were sequentially injected intravenously *via* the right upper limb using a double-barrel high-pressure syringe. Multi-phase dynamic scans were started with a delay of 7 s, separated by 2 s during the arterial phase and 5 s during the venous phase, and volumetric data were obtained for a total of 19 phases, with a total scan time of approximately 60 s.

Image processing was performed as follows (19): Whole-brain CTP volume data were imported into dedicated perfusion post-processing software (4D Perfusion Vitrea fx 6.02, Toshiba, Tochigi Pref., Japan); the MCA was labelled as the input artery, the venous sinus was labelled as the return vein, and a time–density curve was obtained. Pseudocolour maps of four perfusion parameters—cerebral blood flow (CBF), cerebral blood volume (CBV), mean transit time (MTT) of the capillaries and time to peak (TTP)—were generated automatically according to the singular decomposition value.

Setting the region of interest (ROI): The lateral ventricle body level 50 mm above the auditory canthus was selected as the observation level. The ROI was oval and located on the lateral side of the lateral ventricle body; the upper level was level with the anterior horn of the lateral ventricle (frontal angle), the lower level with the posterior horn of the lateral ventricle (occipital angle) and the lateral level with the lower part below the frontotemporal cortex. The ROI of the lesion was delineated manually, and the contralateral corresponding ROI was produced in a mirror image, with the longitudinal fissure as the central axis. The maximum cross-sectional area of each ROI and its four perfusion parameter values were recorded.

The main structure of this region is the corona radiata, which is the sensitive region of reduced blood supply originating from the MCA. The cortical vessels in this region are also commonly used as receptor vessels in STA–MCA anastomosis and are sensitive to changes in CBF during the procedure. At the same time, the selected ROI region was not influenced by the structure of the cerebral ventricle, lateral cleft blood vessels or smoke vessels at the skull base on the CTP parameters or by the posterior cerebral artery, anterior choroid artery or anterior cerebral artery. Two experienced technicians blinded to the patients’ clinical information evaluated the hemodynamic data independently.

### Postoperative evaluation

Each patient had a follow-up visit 6 months following discharge. Postoperative complications during follow-up, such as stroke events, epilepsy and wound infections, were assessed and recorded. Computed tomography perfusion and DSA were performed preoperatively and at 6 months after surgery to evaluate early bypass function. The ROIs were identified for measurement and analysis based on abnormal perfusion areas obtained from the examinations. The perfusion parameters of CBF, CBV, MTT and TTP were obtained and analysed. The clinical status of each patient was defined as improved using the modified Rankin scale (mRS) [The mRS is a clinician-reported measure of global disability comprising seven grades from 0 to 6, with 0 corresponding to no symptoms and 6 corresponding to death (20).]. The mRS was measured preoperatively and at 1 week and 6 months after surgery.

### Statistical analysis

The data in this study were analysed using SPSS (version 22.0, IBM Corp. Silicon Valley, CA, USA) software. The quantitative data were described as mean ± standard deviation and compared using a *t* test or a one-way analysis of variance followed by least-significant-difference tests. The categorical data were described as numbers (percentages). A value of *p* < 0.05 was considered statistically significant.

## Results

A total of 65 patients, with a mean age of 40.97 ± 10.32 years, who met the inclusion criteria were enrolled in this study. Among them, 38 (58.46%) patients were male and 27 (41.54%) were female. Most of the patients (60/65, 92.31%) were ischaemic types, and the symptomatic hemisphere was treated first due to a symptomatic stroke or TIA. Forty-nine (75.38%), 5 (7.69%), 3 (4.62%) and 8 (12.31%) patients received bypass due to stroke, TIA, cerebral haemorrhage and headache, respectively. The majority of patients (62/65, 95.38%) received unilateral surgery, while 3 patients (4.62%) received bilateral revascularisation procedures. The mean operative time was 260.18 ± 37.06 min, ranging from 175 to 365 min. The preoperative Suzuki staging results indicated that there were more patients in stages 3 and 4 and that the Moyamoya vessels had increased gradually in most patients. The postoperative Matsushima grade indicated that most of the patients were grades A and B and that the vascular status of the patients had recovered well. The characteristics of the patients are presented in Table 1.

**Table 1:** The characteristics of the patients enrolled (*n* = 65).

Characteristics	Results
**Age, years**	
Mean	40.97 ± 10.32
Range	28–70
**Sex, *n* (%)**	
Male	38 (58.46)
Female	27 (41.54)
**Preoperative symptoms, *n* (%)**	
Stroke	49 (75.38)
TIA	5 (7.69)
Cerebral hemorrhage	3 (4.62)
Headache	8 (12.31)
**Locations**	
Unilateral	62 (95.38)
Bilateral	3 (4.62)
**Mean operative time, min**	
Mean	260.18 ± 37.06
Range	175.00–365.00
**Suzuki stage preoperatively**	
I	0
II	2
III	36
IV	27
V	0
VI	0
**Postoperative Matsushima grading**	
A	18
B	36
C	9
Not rechecked	2

**Figure 2:**
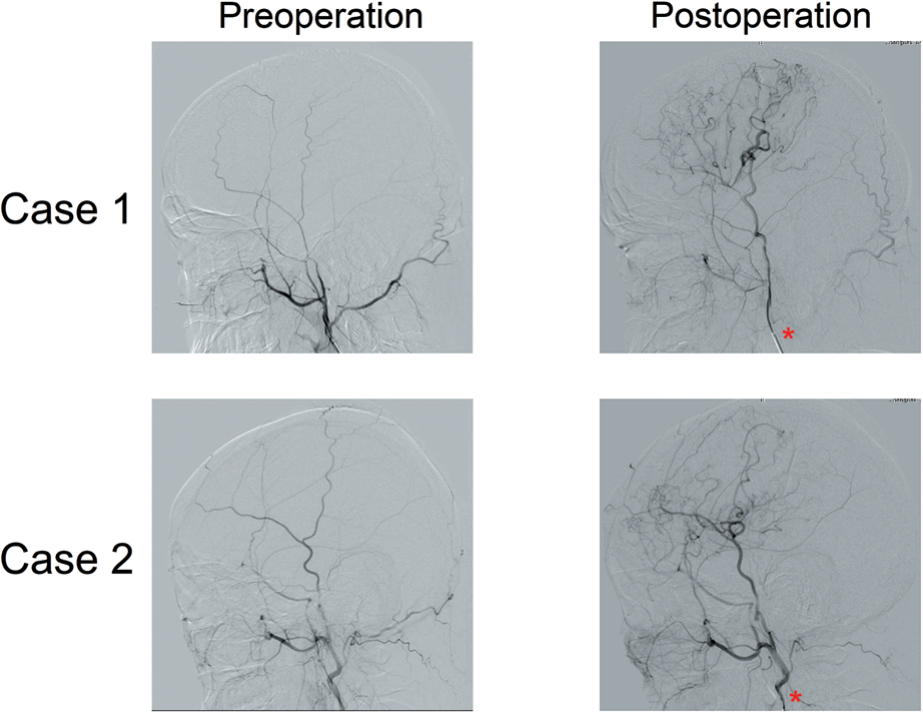
Representative images of DSA before and after surgery. At 6 months after surgery, the symptomatic hemispheres were found to have a functioning extra-intracranial bypass. The specific contrast agent injection site (external carotid artery) is indicated by a red asterisk.

**Figure 3:**
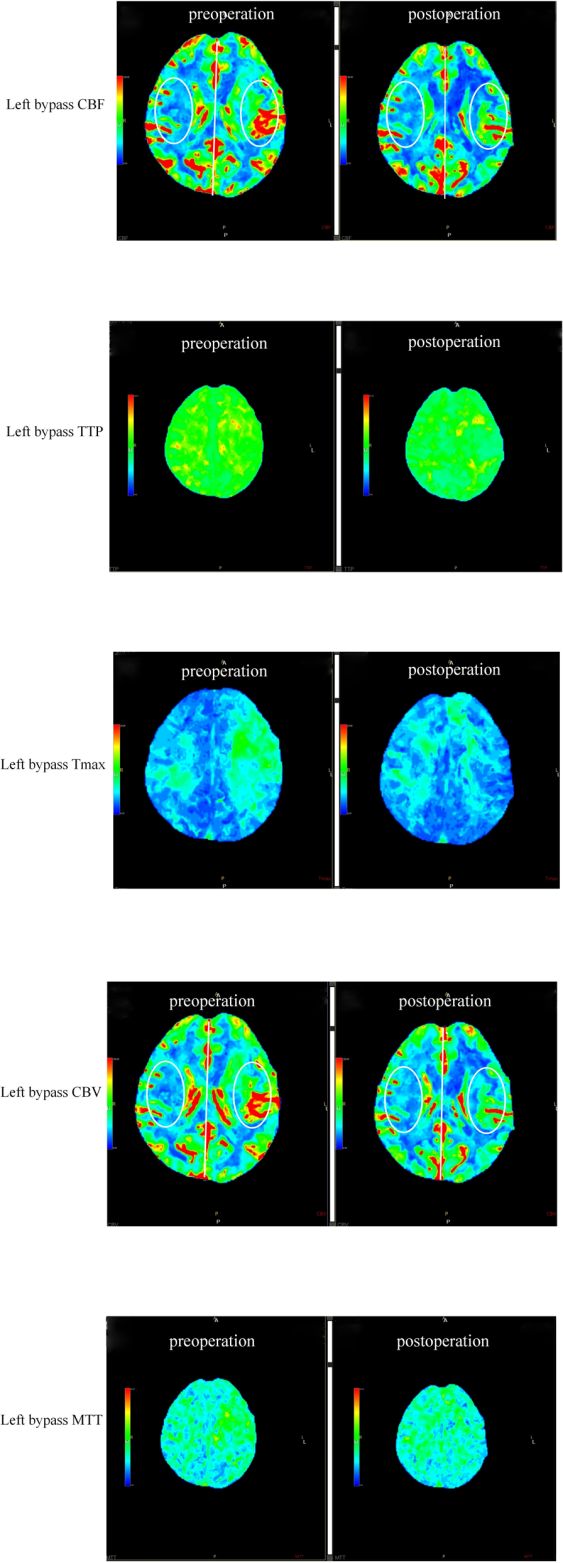
Representative images of CTPs before and after surgery. Images with five parameters: CBF, TTP, Tmax, CBV, and MTT.

**Figure 4:**
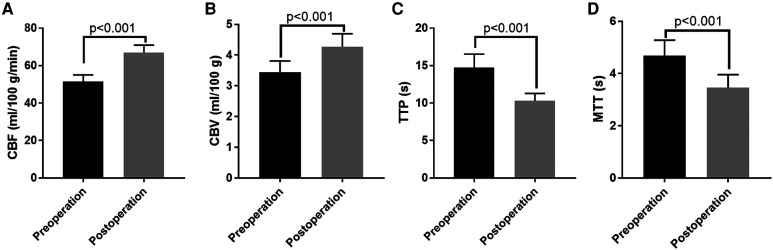
The comparisons of the perfusion parameters after CT perfusion imaging of patients before and after surgery. (**A**) The comparison of CBF; (**B**) The comparison of CBV; (**C**) The comparison of TTP; (**D**) The comparison of MTT. CBF, cerebral blood flow; CBV, cerebral blood volume; MTT, mean transit time; TTP, time to peak.

Among the 65 enrolled patients, postoperative complications were observed in 5 (7.69%) cases, with 2 cases of dysphasia, 2 cases of new cerebral infarction followed by cerebral haemorrhage (both patients were ischaemic types) and 1 case of seizure. Six months after surgery, 66 out of 68 hemispheres were found to have a functioning extra-intracranial bypass (Figures 2, 3), and the patency rate was 97.06%. To evaluate the effect of bypass surgery on CBF perfusion, the perfusion parameters of CBF, CBV, MTT and TTP after CTP imaging were analysed. As shown in Figure 4, compared with the preoperative measurements, both CBF and CBV increased significantly, while MTT and TTP decreased significantly 6 months after surgery (*p* < 0.001). Regarding the prognosis of patients with complications, two cases with speech disorders recovered completely. The patient with epilepsy (*n* = 1) continued taking oral anti-epileptic drugs 6 months after surgery, and there was no recurrence of seizures. Of the two cases with new cerebral infarction followed by cerebral haemorrhage, one was in a vegetative living condition and had a National Institute of Health stroke scale (NIHSS) score of 37 points 6 months after surgery; the other case had hemiplegia and partial aphasia, with a NIHSS score of 25 points 6 months after surgery.

**Figure 5:**
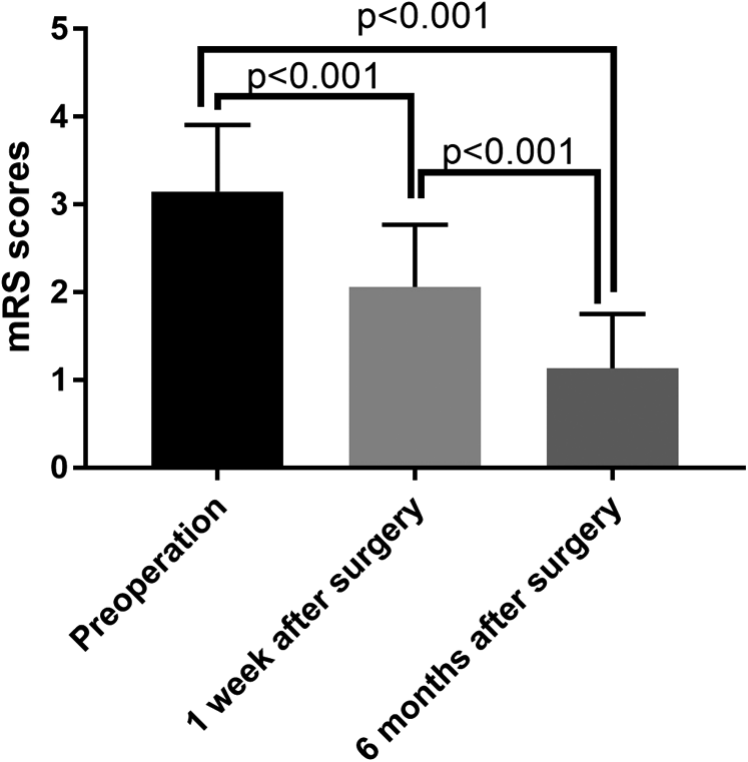
The comparison of mRS scores before and after surgery. mRS, modified Rankin Scale.

Meanwhile, mRS scores of 0–2 (favourable outcomes) were observed in 95.38% (62/65) of the patients 6 months after surgery, while the mRS scores measured 1 week after surgery (2.06 ± 0.71 vs. 3.15 ± 0.76, *p* < 0.001) or 6 months after surgery (1.13 ± 0.62 vs. 3.15 ± 0.76, *p* < 0.001) were much lower than those measured preoperatively (Figure 5). Furthermore, compared with those obtained 1 week after surgery, the mRS scores decreased significantly 6 months after surgery (*p* < 0.001).

## Discussion

In the present study, we evaluated the efficacy and safety of a combined revascularisation strategy (STA–MCA + EMS) in Chinese adult patients with MMD. The main findings can be summarised as follows: (1) The rates of postoperative complications and patency were 7.69% and 97.06%, respectively. (2) Both the CBF and CBV increased significantly, while the MTT and TTP decreased after surgery. (3) The short-term and long-term postoperative mRS scores were much lower than those measured preoperatively.

To the best of our knowledge, this is the first study to demonstrate that STA–MCA + EMS bypass is a feasible and safe method for Chinese patients with MMD, potentially significantly improving CBF perfusion and clinical symptoms without increased postoperative complications. Our study provides the latest evidence for the use of this approach in the Chinese population and is expected to further change clinical decision making and provide benefits for prognosis. However, further direct comparisons between the combined bypass and direct bypass methods are needed.

Surgical revascularisation is the standard treatment for MMD. However, the optimal revascularisation strategy for MMD has not yet been determined. The STA–MCA bypass method entails direct revascularisation surgery, which can provide immediate flow augmentation *via* the anastomosis vessels (12, 21). The method can also restore CBF in the clinically leading hemisphere immediately following surgery, thereby reducing the risk of new cerebral infarction (5). Meanwhile, EMS is an indirect revascularisation surgery for patients with MMD that uses the temporalis muscle as the donor organ (22). This method can stimulate collateral formations and improve impaired haemodynamics (23). Furthermore, EMS has been reported to play an important role in long-term revascularisation (23). However, EMS requires at least 3 months for neo-angiogenesis between vascularised extracranial tissues and the brain cortex. Thus, the benefit of EMS is limited for adult patients with symptomatic MMD due to its disadvantage of delayed blood-supply restoration (12).

In our institution, we prefer to perform combined bypasses (STA–MCA + EMS) rather than single use in adult patients with MMD. The combined revascularisation strategy has the advantages of both direct and indirect surgeries, and several previous studies have recommended the combined strategy. For instance, Czabanka et al. (5) found that STA–MCA + EMS had a stronger ability to restore cerebrovascular reserve than EMS alone, while Bot et al. (13) reviewed a series of infant (≤3 years of age) patients with MMD who were treated with STA–MCA + EMS and demonstrated that this combined strategy was feasible and safe. Elsewhere, Imai et al. (11) analysed the importance of direct, indirect and combined revascularisation strategies for patients with MMD and found that EMS had the advantage of promoting revascularisation in the ischaemic area, thereby playing an important role in combined STA–MCA + EMS surgery.

Similar to these previous studies, we also found that STA–MCA + EMS surgery had a good extra-intracranial bypass patency rate (97.06%) 6 months after surgery. Furthermore, the perfusion parameters indicated that the CBF and CBV increased significantly, while the MTT and TTP decreased 6 months after STA–MCA + EMS surgery. These results indicate that STA–MCA + EMS surgery might significantly improve CBF perfusion. Corresponding to the improvement in CBF perfusion, clinical symptoms might also improve after surgery, and the headache and cerebral ischaemia symptoms of patients might be alleviated. The mRS scores measured 1 week after surgery or 6 months after surgery were lower than those measured preoperatively. One possible reason for this is that the EMS stimulated collateral formation and improved the blood supply. The mRS score reflects the cerebral function, and thus, its improvement may have resulted from enhanced blood perfusion. Previous studies have demonstrated a significant correlation between the mRS score and blood distribution in several neurological deficits, including subarachnoid haemorrhage (24), acute ischemic stroke (25) and symptomatic intracerebral haemorrhage (26). In addition, compared with the mRS score 1 week after surgery, the mRS scores decreased 6 months after surgery. A possible reason is the prolonged persistent increase in blood supply induced by EMS. In general, the mRS score after combined bypass surgery was lower than that before surgery. The reason for this may be related to how, during STA–MCA + EMS surgery, the STA–MCA bypass, in conjunction with the direct and indirect bypass-related advantages of study (27), effectively rapidly improved the local blood supply to the brain while maximising the use of the external carotid artery's blood supply (28). The EMS applied vascular-rich muscle tissue to the cortical surface of the brain, stimulating the cerebral cortex and promoting the production of new blood vessels to effectively establish collateral circulation (29). The two techniques complemented each other and compensated for the limitations of single use, meaning that patients with MMD can obtain better efficacy (30).

Previous studies have demonstrated that the indirect bypass method had low incidence rates of postoperative complications, such as neurological deficits and seizures (12). However, due to its disadvantage of delayed blood-supply restoration, those patients who received EMS alone had a higher risk of cerebral infarction (5). It has been reported that the 5-year cumulative risk of recurrent stroke ranged from 6% to 15% for patients who received EMS alone (12). Our postoperative cerebral infarction risk was relatively low, with only two patients experiencing a new symptomatic cerebral infarction. This phenomenon may be due to the immediate revascularisation of STA–MCA bypass without the biological delay associated with EMS bypass. The risk of other postoperative complications of STA–MCA bypass appeared to be higher than with EMS bypass. In this study, postoperative complications were observed in 7.69% of patients, similar to the historical data for STA–MCA alone (12, 21). Collectively, the safety data obtained in this study indicated that the combined revascularisation strategy might not increase the risk of postoperative complications.

The present study has several limitations. First, this study was conducted in a single centre with a small sample size. A multi-centre study with a larger sample size is needed to confirm the conclusions of this research. Second, the follow-up duration of this study was only 6 months, which is relatively short. The long-term outcomes of reperfusion from the bypass procedure should be evaluated. Third, this study included only one treatment arm. We did not collect enough data to compare the efficacy of combined surgery with direct/indirect revascularisation surgery alone. In the future, further studies are necessary to compare this combined strategy with direct or indirect strategies. Fourth, as EMS is the main procedure performed in our centre, only EMS bypass was performed as the indirect bypass procedure in the combined revascularisation surgery in the present study. Therefore, the effect of STA–MCA combined with other indirect procedures, such as EDAS, should be included in future research. Finally, there was a lack of preoperative evaluation of choroidal artery and medullary artery perforation, which might be related to haemorrhagic risk during follow-up. Subsequent efforts should be made to investigate the relationship between these arteries and adverse events after surgery.

## Conclusion

In the present study, it was found that direct STA–MCA bypass in combination with indirect EMS bypass is a feasible and safe method for Chinese adult patients with MMD. The method can significantly improve CBF perfusion and clinical symptoms without increasing postoperative complications.

## Data Availability

The original contributions presented in the study are included in the article/Supplementary Material, further inquiries can be directed to the corresponding author.
